# Analysis of *Drosophila melanogaster* testis transcriptome

**DOI:** 10.1186/s12864-018-5085-z

**Published:** 2018-09-24

**Authors:** Viktor Vedelek, László Bodai, Gábor Grézal, Bence Kovács, Imre M. Boros, Barbara Laurinyecz, Rita Sinka

**Affiliations:** 1 Department of Genetics, University of Szeged, Szeged, Hungary; 2 Department of Biochemistry and Molecular Biology, University of Szeged, Szeged, Hungary

**Keywords:** Drosophila, Transcriptome, RNA sequencing, Testis, Spermatogenesis

## Abstract

**Background:**

The formation of matured and individual sperm involves a series of molecular and spectacular morphological changes of the developing cysts in *Drosophila melanogaster* testis. Recent advances in RNA Sequencing (RNA-Seq) technology help us to understand the complexity of eukaryotic transcriptomes by dissecting different tissues and developmental stages of organisms. To gain a better understanding of cellular differentiation of spermatogenesis, we applied RNA-Seq to analyse the testis-specific transcriptome, including coding and non-coding genes.

**Results:**

We isolated three different parts of the wild-type testis by dissecting and cutting the different regions: 1.) the apical region, which contains stem cells and developing spermatocytes 2.) the middle region, with enrichment of meiotic cysts 3.) the basal region, which contains elongated post-meiotic cysts with spermatids. Total RNA was isolated from each region and analysed by next-generation sequencing. We collected data from the annotated 17412 Drosophila genes and identified 5381 genes with significant transcript accumulation differences between the regions, representing the main stages of spermatogenesis. We demonstrated for the first time the presence and region specific distribution of 2061 lncRNAs in testis, with 203 significant differences. Using the available modENCODE RNA-Seq data, we determined the tissue specificity indices of Drosophila genes. Combining the indices with our results, we identified genes with region-specific enrichment in testis.

**Conclusion:**

By multiple analyses of our results and integrating existing knowledge about *Drosophila melanogaster* spermatogenesis to our dataset, we were able to describe transcript composition of different regions of Drosophila testis, including several stage-specific transcripts. We present searchable visualizations that can facilitate the identification of new components that play role in the organisation and composition of different stages of spermatogenesis, including the less known, but complex regulation of post-meiotic stages.

**Electronic supplementary material:**

The online version of this article (10.1186/s12864-018-5085-z) contains supplementary material, which is available to authorized users.

## Background

A Drosophila testis is a blind-ended tube, where consecutive stages of spermatogenesis are presented in the developmental order from the apical end to the basal end. The apical tip of the testis is enriched with somatic hub cells, germ-line stem cells enclosed by cyst progenitor cells and the dividing gonialblast cells. Four synchronous divisions result in cysts with 16 primary spermatocytes. Two somatic cyst cells support the germline cells throughout the entire spermatogenesis process. Meiotic cysts, consisting of 64 haploid spermatids in each one, are found in the middle region of the testis. The basal region contains mainly the post-meiotic elongated, individualizing cysts. After meiosis, the cells change shape and size dramatically and develop specialized cellular organelles, such as the basal body, axoneme and acrosome [1]. Most of the gene products necessary for late stages of spermatogenesis are transcribed before meiosis, including genes contributing to the formation of the sperm head and tail, such as the cytoskeletal components, genes of mitochondrial enzymes, protamines and also enzymes responsible for individualisation. Actin-based structures, known as investment cones, assemble around the 64 elongated spermatid nuclei, move together towards the distal end of the cyst during individualization, and most of the cytoplasm of the spermatids is simultaneously degraded and individual plasma membranes are formed around each elongated spermatid [2, 3].

Enzymes and structural components of the mitochondria are synthesized mainly from the nuclear genome. Mitochondrial enzymes frequently contain duplicates with testis-biased expression patterns for one of them, based on microarray analysis of Drosophila males and females [4, 5]. Spermatid mitochondria aggregate, fuse and form the Nebenkern in round spermatids [2]. During elongation, the Nebenkern unfurls and establishes two mitochondrial derivatives which elongate in the entire 1.8 mm length of the spermatid tail near the axoneme. The major derivative accumulates paracrystalline material and the minor one reduces its volume [6]. However, the molecular composition and proteins responsible for the regulation of changes in mitochondria during spermatogenesis are not fully understood.

A testis region-specific microarray experiment proved the presence of X chromosome-linked gene inactivation during male meiosis and suggested that meiotic sex chromosome inactivation could be a general mechanism which contributes to the evolution of some testis-biased genes [7], however, the region specific enrichment of gene products were not analysed in detail. Developmental processes associated with germ cell formation are likely to involve novel genes and there is growing evidence that indeed many ubiquitously expressed genes have testis-specific paralogues [8]. These gene duplication events are typical in metabolic enzymes located mainly in the mitochondria, proteasome subunits and also in the case of several cytoskeletal elements [9]. RNA-Seq is a versatile method for measuring transcript levels, identifying alternative splice variants and a reliable platform to identifying polyA containing lncRNAs.

Here we report the transcriptomic profiling of functionally and anatomically different parts of the *Drosophila melanogaster* testis using RNA-Seq. Our work highlights the molecular composition of different regions of Drosophila testis. By integrating our dataset and the publicly available databases, we determined and visualized expression patterns of functionally related genes and correlated their known and predicted functions to different parts of the testis.

## Results

### Transcriptome analysis of testis using RNA-Seq

To gain a better understanding of cellular differentiation during spermatogenesis, we decided to compare transcript composition of different parts of the Drosophila testis. We cut testes into three parts: apical, middle and basal regions, which represent the proceeding stages of spermatogenesis (Additional file 1: Figure S1A) [7]. The apical region contains the spermatogonial stages represented by mitotically dividing cells; the middle region of the testis is enriched with meiotic spermatocytes and the basal region is filled with transcriptionally inactive elongated spermatids. Two cyst cells, of somatic origin, cover the 64 elongated spermatids and contribute to the transcriptome of the examined regions.

We performed RNA Sequencing of poly(A)^+^ RNA in biological duplicates from dissected testis regions. We used the Illumina MiSeq platform as described in the Materials and Methods to sequence gene products for each region. Transcriptome assembly and differential transcript accumulation analysis of RNA-Seq was done with Cuffdiff program. Using Fragments Per Kilobase per Million mapped reads (FPKM) values of individual genes, we compared transcript levels between apical, middle and basal regions of the testis.

Previously, Vibranovski et al. published transcriptome data containing 18082 records, based on GeneChip® Drosophila Genome 2.0 Array (Affymetrix) experiments. The probes were 25 nt length oligos designed based on the 3.1 *D. melanogaster* genome release. The microarray experiments reported of 13150 genes [7]. The present RNA-Seq data were analysed using the Dmr6.05 *D. melanogaster* reference genome and corresponding transcript annotation that contains 33869 records of transcripts and 17412 gene records.

**Fig. 1:**
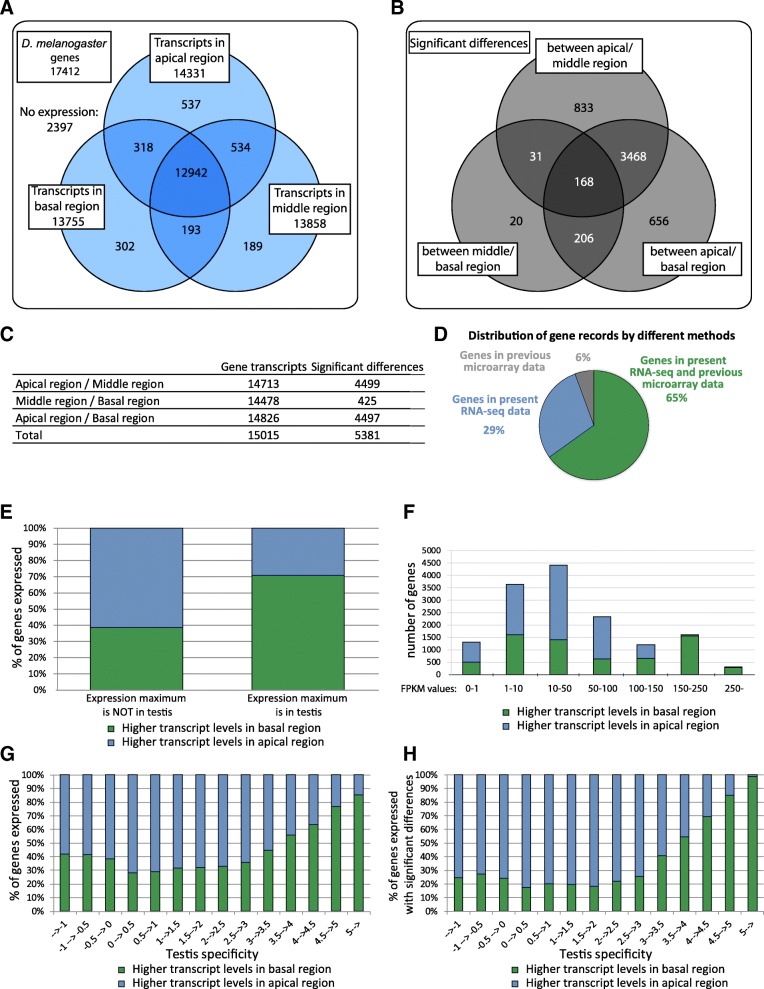
Distribution of transcripts in different region of testis and analysis of the RNA-Seq results. **a** Distribution of *Drosophila melanogaster* transcripts by RNA-Seq. **b** Number of genes showing significant differences (FDR-corrected *p*-value< 0.05) in transcript level between different region of testis. **c** The combined number of transcripts and the significant differences (FDR-corrected *p*-value< 0.05) between different region of testis. **d** Comparison of the previous microarray data [7] with present RNA-seq data. **e** Distribution of genes with lower or higher transcript levels in the basal testicular regions compared to the apical region. The comparison was based on the tissue where their expression maximum is defined by modENCODE database [56]. **f** Distribution of transcript level differences in basal region compared to the apical region as a function of gene expression levels. **g** Distribution of differences in transcript levels along the apical-basal axis of the testis as a function of tissue specificity index. **h** Distribution of genes with statistically significant transcript levels along the apical-basal axis of the testis as a function of tissue specificity index

We identified transcription profiles for 15015 Drosophila genes in different regions. 2397 of the known Drosophila genes showed no measurable expression in any examined region. This could be due to the fact that these genes are expressed at very low levels or their expression is restricted to a different tissue. We identified transcripts of 14331 genes in the apical region, 13858 genes in the middle region and 13755 in the basal region of the testis (Fig. 1 a). Transcript accumulation of 12942 genes was detected in all three regions, and 537 transcripts were present exclusively in the apical region, while 189 were only in the meiotic region and 302 only in the post-meiotic region (Fig. 1 a). We also analysed the transcript accumulation of 2061 lncRNAs in distinct stages of spermatogenesis, which were not present in the microarray analysis platform [7]. We determined the transcript accumulation pattern for each gene and filtered genes that showed significant transcript level differences (*p*-value< 0.05) between regions based on Cuffdiff analyses (Additional file 2). 5831 genes show significantly higher or lower transcript levels between different regions (Fig. 1 b, c). We found 4499 significant differences between the apical and middle regions, including 833 differences, which are unique for these two regions. Apically-enriched transcripts could serve the protein pool responsible for germline differentiation and mitotic divisions of the spermatogonial cells. We found 20 unique differences out of the 425 significant ones between meiotic middle and post-meiotic basal regions and 656 unique differences out of 4497 significant differences between apical and basal regions (Fig. 1 b, c). Transcripts of protein-coding genes that are enriched in the middle or basal region after translation could direct nuclear and cyst elongation, including axonemal assembly and mitochondrial elongation. Transcripts with basal accumulation are products of genes which are involved in the individualisation of the cysts and contribute to the structure of the mature sperm.

### Evaluation of the testis transcriptome

To test the reliability of our method, we compared the microarray experiments of Vibranovski et al. with our RNA Sequencing results [7]. First, we converted the probe IDs of the microarray data to FlyBase IDs. We compared the RNA-Seq and the converted microarray datasets and found 65% overlap between the two methods, 6% of the genes were represented only in the microarray experiment and 29% of the annotated genes were represented only in the RNA-Seq data, probably due to the higher sensitivity of RNA-Seq method and the more precise annotation of the *D. melanogaster* genome (Fig. 1 d). We also performed a region specific comparison of normalized expression values by selecting 3015 male-specific genes from the Sebida database, which contains genes with sex-biased expression patterns [10]. We observed good correlation between the two datasets in every testis region with the highest correlation in the basal region (Additional file 1: Figure S1B), proving the reliability of the RNA-Seq results.

For further analysis, differences of transcript levels, based on our RNA-Seq results, between the apical and the basal region of testis were compared. We used the modENCODE tissue expression database to filter our dataset for genes with maximal expression in testis. Our results show that for genes with maximal expression in the testis, transcript levels are generally higher (70.8%), while in the case of genes that are expressed in other tissues, transcript levels are more likely (61.3%) lower towards the basal region of the testis (Fig. 1 e). By comparing the FPKM values in our dataset, we were able to group genes from minimal to very high expression. This analysis revealed that genes with low or moderate expression generally showed decreased accumulation, while genes with high expression, show higher transcript accumulation in the basal region compared to the apical region (Fig. 1 f).

A comparative tissue-specific gene expression analysis between *C. elegans* and *D. melanogaster* was reported by Li et al. in 2014 [11]. They determined the tissue specificity of each gene using the RNA-Seq results from the modENCODE database. Using their formula, we determined the tissue specificity of *Drosophila melanogaster* genes based on the modENCODE tissue expression database and identified the testis-enriched genes (Additional file 2). Higher tissue specificity scores represent testis-specific enrichment of transcripts compared to other tissues. The value of the index varies between − 2.52 and 5.2. The negative values represent the underrepresentation of transcripts in testis, the lower values represent genes expressed in multiple tissues or ubiquitously. Genes with an index higher than 4 are highly enriched in testis (Additional file 2). High testis specificity does not necessarily mean high expression levels. If there is a minimal expression level in the testis, but no expression in other tissues, it could result in a high tissue specificity index, such as in the case of *Hr83*, *CG3669*, and *CR46206* (Additional file 2). The transcript level of genes with lower testis specificity generally decreases, while genes with higher testis specificity generally elevated along the apical-basal axis of the testis (Fig. 1 g).

Exclusive analysis of the significant transcript level differences between the apical and basal regions, indicated that the majority of transcripts with high testis specificity show transcript accumulation towards the basal region (Fig. 1 h).

We analysed the transcript accumulation of genes based on their tissue specificity (Additional file 1: Figure S1C). Our results show that genes with higher testis specificity indices generally have higher transcript levels with a substantial Pearson correlation value of 0.60. Investigating only the significant differences between different regions, we observed a similar tendency with a Pearson correlation value of 0.70 (Additional file 1: Figure S1D).

To test the spatial distribution of transcripts that contribute to the formation of the *Drosophila melanogaster* sperm proteome (DmSP), we collected four sets of proteomic data and filtered out 263 proteins that appeared at least three times in the DmSP datasets [12, 13]. We visualised the transcript level log2 fold changes of the 263 genes corresponding to these proteins between the apical and basal regions based on the testis specificity indices of the genes (Additional file 1: Figure S1E). The graph shows that the majority of these genes are testis-enriched with a median tissue specificity value of 4.35 (average: 2.77) and 206 genes have higher transcript levels in later stages (transcript levels of 46 genes are lower). This result suggests that the majority of transcripts accumulating in the late stages are responsible for the formation and function of the mature sperm.

Analysis of our data showed that testis-enriched genes generally have higher transcript levels and accumulate in the later stages of spermatogenesis. We need to highlight the fact that in spite of these tendencies, there are ubiquitously expressed genes with high transcript levels in later stages and also with considerable transcript level differences between the apical and basal regions. Nevertheless, our data suggest that the formation of the unique structures and molecular composition of sperm relies on various testis-specific gene products, accumulating in the later stages of spermatogenesis.

### Gene ontology (GO) analysis of RNA-Seq data

GO term enrichment analysis can highlight the biological relevance of sequencing data. We used the GOrilla online analysis tools to search the enriched GO terms in our dataset [14]. GOrilla offers two options to analyse a dataset, the first mode is able to search GO enrichment at the top of a ranked list. We generated these ranked lists based on the testis tissue specificity index and on the transcript levels in different developmental stages. We conducted the analysis in all GO categories (biological process, molecular function, cellular component), and compared the corresponding datasets to each other (Additional file 3). As expected, there is an overrepresentation of cell cycle-related proteins and components of the transcription apparatus in the apical region, where the mitotically dividing spermatocytes are present. However, in the basal region, we found an enrichment of GO terms corresponding to cytoskeletal, nuclear and mitochondrial proteins that contribute to the formation of mature sperm. This kind of analysis highlights some interesting aspects of our data; however, this approach omits genes with important functions but low transcript levels, such as the regulator genes of early spermatogenesis. For example, *bam* expression is necessary for the differentiation of gonialblast cells, however, in the ranked list, *bam* expression is at the 7174th position out of 17409, which means that *bam* is not in the top 40% of the list, despite its important function in cell fate regulation (Additional file 3).

GOrilla provides another analysis mode, where a subset of genes can be analysed based on a background list. We could generate gene lists based on chosen properties of our dataset, for example, using a list of genes with significantly lower transcript levels in later stages, so we can detect more specific enrichments in early stages. We conducted our analysis in all GO categories, focusing on genes with significant transcript level differences between the apical and basal regions (Additional file 3). We also conducted our analysis based on resolved testis specificity index (Additional file 3). Thus, we obtained an overall picture of GO term enrichment. Our analysis confirmed that GO terms related to early development are related to ubiquitously expressed genes with decreasing transcript levels (nuclear components, intracellular parts, RNA processing, etc). GO terms related to later stages, are more likely testis-enriched and have higher transcript levels (mitochondrial parts, pyruvate kinase activity, ATP metabolic process, etc). GO analysis highlighted that metabolic pathways and mitochondria have many testis-specific elements that are expressed in later stages (Additional file 3). This corresponds with the crucial role of mitochondria during spermatogenesis and their function in mature sperm.

### Visualization of the results by Cytoscape and validation of the RNA-Seq

To further visualize and analyse our dataset, we generated multiple graph-based networks with Cytoscape [15]. We created a network based on the FlyBase “Gene Group Reports” database, which brings together genes/gene products that are members of the same biological group, including functional groups, protein complexes and gene families in a searchable format (Additional file 4). We extracted sub-networks, which exemplify some of the most studied protein families implicated in different aspects of spermatogenesis (Fig. 3-7, Additional file 1: Figure S2–4). Furthermore, we present two networks, based on the KEGG metabolic pathways, a custom build network of the mitochondrial respiratory chain elements, and a representation of long non-coding RNAs (Fig. 8 Additional file 1: Figure S5–7). In the presented networks and sub-networks we used consistent labelling methods. Node size represents testis enrichment, where larger nodes indicate testis-enriched genes. The border of the gene node symbolises maximum expression values, higher expression values are shown with a thicker border. The shape of the gene node represents the number of significant differences between different regions (apical-middle, apical-basal, middle-basal): diamond-shaped nodes have no significant differences, genes with hexagonal nodes have one significant difference, octagonal have two and circular nodes have three significant differences. The colour of gene nodes corresponds to the log2 fold change of transcript levels between the apical and basal regions. The colour gradient runs from red through white to green, where red nodes represent higher transcript level in the apical, while green nodes symbolise higher transcript level in the basal region. The scale is individually adjusted to the dataset and we included detailed information in the legend of each figure.

**Fig. 2:**
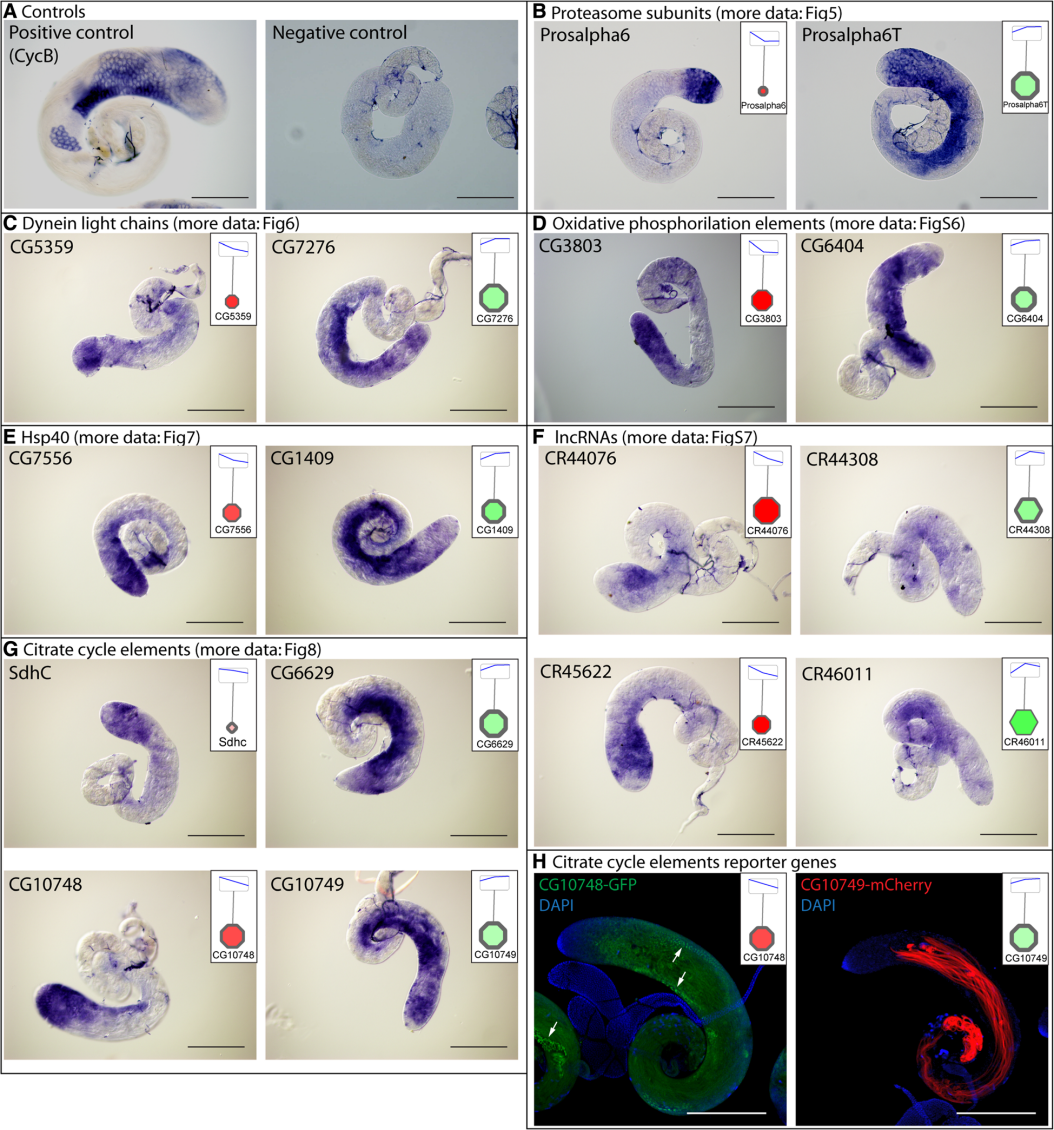
In situ hybridization and reporter genes. **a** Positive control (CycB) and negative control (sense probe) of the in situ hybridization. **b**-**g** Transcript distribution of genes from different gene groups by DIG-RNA in situ hybridization. Insets show the RNA-seq data of the genes visualized by Cytoscape. **h** Expression pattern of the testis-specific malate dehydrogenases, CG10748-GFP and CG10749-mCherry in testis. Arrow points to the nebenkern. Scale bars represent 200 μm

We validated the RNA-Seq results with in situ hybridization by choosing uncharacterized genes with apically or basally enriched transcripts from each main tested gene group (Fig. 2 a-g). We found good correlation between the RNA-Seq results and the transcript distribution by in situ hybridization.

### Ubiquitin proteasome system

The Ubiquitin Proteasome System (UPS) is responsible the regulated degradation of cellular proteins and protein turnover [16]. Components of the UPS are involved in different steps and processes during gametogenesis, including control of germ-line stem cell maintenance, meiosis, reorganization of chromatin structure and individualisation [17–22].

**Fig. 3:**
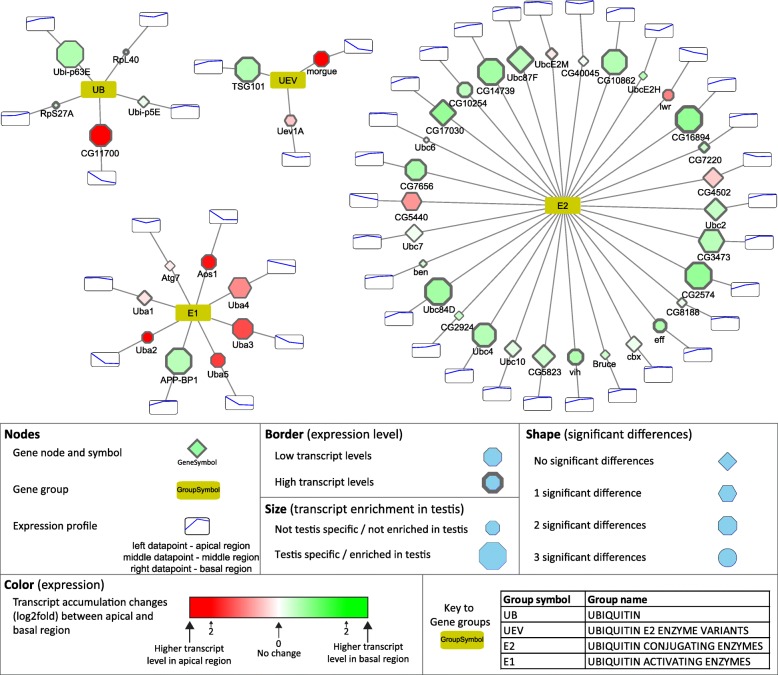
Transcript distribution of the ubiquitin activating E1 and ubiquitin conjugating E2 genes. Transcript differences between apical and post-meiotic regions were visualized by Cytoscape software platform. Bottom part of the figure contains the description of the symbols

Based on the InterPro domain database there are 31 ubiquitin domains containing ubiquitin and ubiquitin-like proteins in *Drosophila*. The transcripts of the five ubiquitin-coding genes, *CG11700*, *RpL40*, *RpS27A*, *Ubi-p5E* and *Ubi-p63E*, show differential distribution in the examined regions of testis. Two of them, *CG11700* and Ubi-p63E, have high and specific transcript accumulation in the testis. The *CG11700* transcript accumulates in the apical and in the middle regions, while Ubi-p63E is enriched in the basal region (Fig. 3). It was shown that *CG11700* is a negative regulator of reproductive processes [23]. Ubi-p63E also has a function during spermatogenesis, in male meiosis and spermatid differentiation, which is consistent with the transcript distribution [24].

The E1 gene group of UPS contains eight genes, with the molecular function of ubiquitin, SUMO, NEDD8, UFM1, or Atg8 activation. Among them, the transcript level of the Nedd8 activating gene (*APP-BP1*) is substantially elevated towards the basal region, however, the precise function of Neddylation during late spermatogenesis has yet to be elucidated (Fig. 3). The number of E2 enzymes is much higher than the E1 enzymes; there are thirty E2s and three ubiquitin E2 enzyme variants (UEV). Their function is to transport the activated ubiquitin to the E3 ubiquitin ligase enzymes. There are eight highly testis-enriched E2 enzymes. There are seven genes, including the two UEVs with higher transcript accumulation in the apical region than in the basal region, while there are twenty-six genes with higher transcript levels in the basal region. Amongst the E2 enzymes, many have elevated transcript levels at the basal end of the testis, which represents the late stages of spermatogenesis (Fig. 3). One of these genes, *effete* was shown to be necessary in telomere function in male meiosis [25]. Another one with basally accumulated transcripts is *dBruce*, and the corresponding protein is part of the CRL3 complex, which has a role in spatial regulation of caspase activity during individualisation [26, 27].

The E3 enzymes exhibit much higher variation than any other group of the UPS system, the E3 group includes 7 subgroups according to the Flybase gene group. Transcripts of the U-Box E3 ligases accumulate mainly in the apical part, where the early stages of cysts are present (Additional file 1: Figure S2). The Roc family of E3 enzymes has a testis-specific ligase, Roc1b, accumulating its transcripts in later stages of spermatogenesis and it is known to be necessary in the final steps of spermatogenesis [28]. The HECT and TRIM families exhibit various transcript accumulation patterns; accumulation of *CG3356* and *bon* are more specific for the apical part, while the testis-enriched transcript of *Ube3a* shows basal accumulation (Additional file 1: Figure S2). Members of the IAP and GOL family show no substantial difference between developmental stages. The RBR family has two testis-enriched members, *CG33144* and *CG12362*, and the second with considerably higher transcript level in the basal region (Additional file 1: Figure S2). Another large group of putative E3 enzymes is the RING finger domain proteins [29]. The RING group contains 137 genes, 33 out of them are highly enriched in testis, moreover 26 have higher transcript accumulation in the basal region. (Additional file 4).

Many of the E3 ligases act as enzyme complexes, like the SKP1, Cullin1, and F-Box proteins in the SCF complex [30]. Potential backbones of E3 enzyme complexes are Cullin proteins. We identified *Cul4*, *Cul5* and *CG11261* transcripts with apical enrichment, while *Cul1*, *Cul2*, and *Cul3* have strong basal accumulation, suggesting their post-meiotic function (Additional file 1: Figure S2). The SkP1family contains five testis-enriched members, three with apical and two with basal transcript accumulation (Additional file 1: Figure S2). Members of the F-box family are components of large E3 ligase complexes, where they are responsible for the substrate specificity of the complexes [31]. There are several testis-enriched F-box transcripts with apical and basal accumulation, suggesting that ubiquitination is tightly regulated during the stages of spermatogenesis (Additional file 1: Figure S2). One known example is *ntc*, which is involved in the substrate specificity of the SCF complex during individualisation [32].

**Fig. 4:**
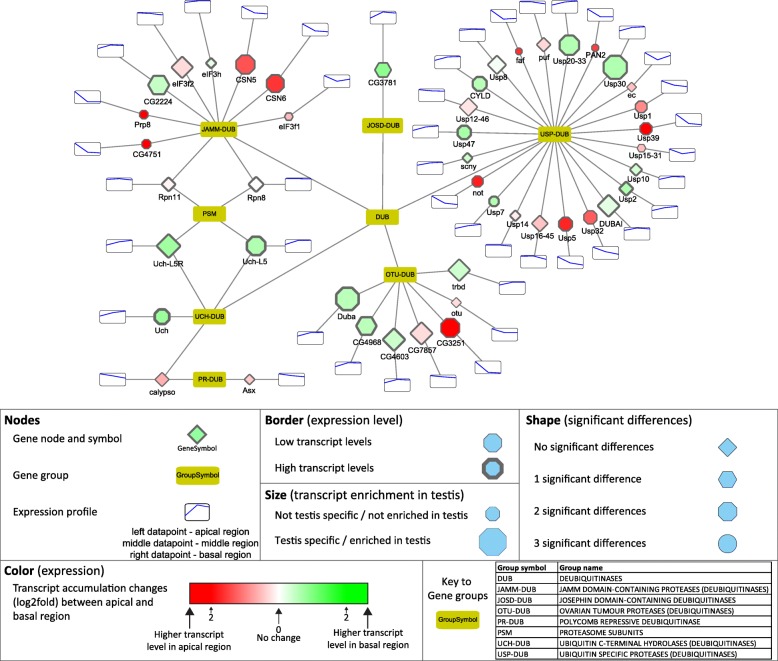
Distribution of transcripts of the deubiquitinating enzymes visualized by Cytoscape

More and more evidence suggests that deubiquitination, the conjugated ubiquitin recycling, is as important as ubiquitination during spermatogenesis [21]. Many of the deubiquitinases (DUB) show significant differences in their transcript accumulation levels between different spermatogenic stages. Almost every group contains testis-enriched variants, both with early and late transcript accumulation (Fig. 4). From the OTU family of DUBs, *CG4968*, *Duba*, *CG4603,* and *trbd* have higher transcript level in the basal region, while *CG3251* is higher in the apical region. OTU and CSN complexes were shown to regulate Bam level in balancing stem cell self-renewal and differentiation in the female germline [19, 33]. *CG3251* is a testis-specific version of the OTU-DUB family, with strong accumulation in the apical region of the testis, where Bam activity is present (Fig. 4). Our data also identified transcripts of the Cop9 signalosome genes, *CSN5* and *CSN6,* with strong transcript enrichment in early stages, supporting their known function in early differentiation [34]. *Duba*, which has transcript accumulation in the basal region, where elongation takes place, was shown to have an important function in non-apoptotic caspase regulation during late spermatogenesis [35].

**Fig. 5:**
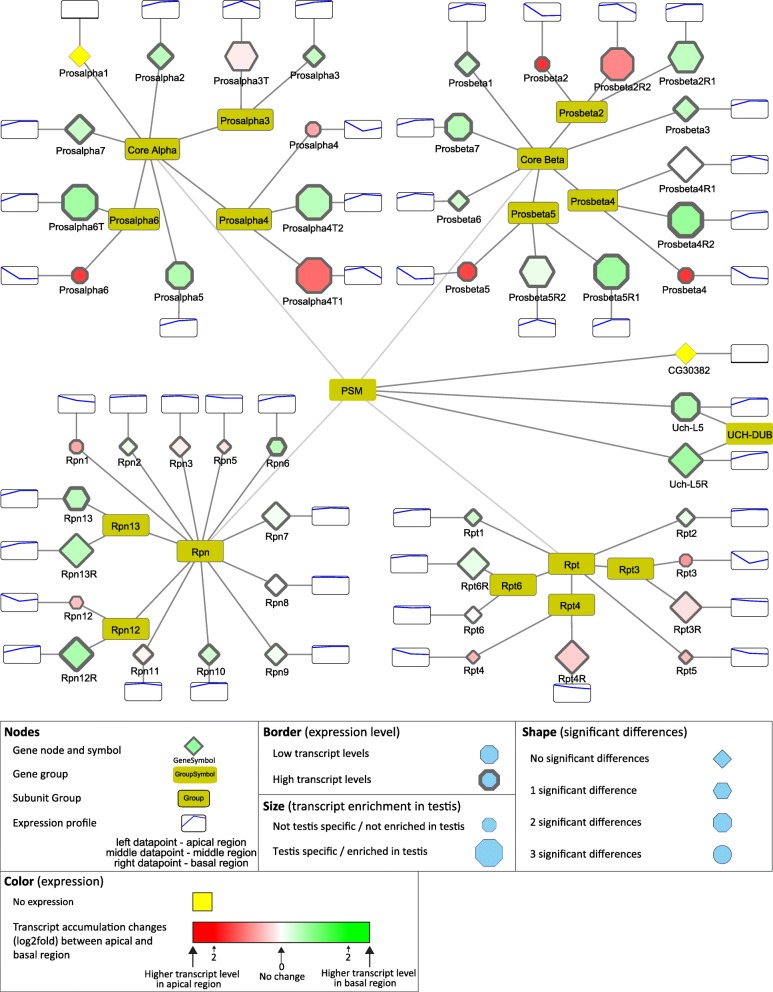
Distribution of transcripts of the 26S proteasome visualized by Cytoscape. The 20S core proteasome is composed of core Alpha and core Beta subunits and the 19S regulatory proteasome are built from Rpn and Rpt subunits

The USP and UCHL family of DUB proteins are represented by several members with considerable transcript accumulation either in the apical or basal regions of the testis. Homologues of USP2, USP8, USP14, UCH-L3, UCH-L5, and CYLD were shown to function in various stages of mammalian spermatogenesis and contribute to gonocyte recruitment, cell cycle progression, regulation of the meiotic phase, spermiogenesis, acrosome biogenesis, and germ cell apoptosis [21]. Usp30 is a testis-enriched DUB with high transcript accumulation in the basal region of the testis, where the post-meiotic spermatids are enriched (Fig. 4). The effector element of the UPS system is the proteasome. The 26S proteasome consists of two main sub-complexes, the 20S core subunit and the 19S regulatory subunit. Testis-specific paralogues of the proteasomal subunits were shown to exist in *Drosophila melanogaster* [36, 37]. Both the core and the regulatory particles have testis-specific counterparts. We found that transcripts of testis-specific alpha and beta core members and the regulatory subunits are enriched mainly in the basal region, suggesting important functions in late spermatogenesis events such as histone-to-protamine transition or individualisation (Fig. 5). We tested the RNA localization of *Prosalpha6* and *Prosalpha6T* by in situ hybridization and proved that it coincides with the published localization of the proteins, suggesting that the RNA localization established in our experiments indeed could reflect the region-specific distribution of the proteins (Fig. 2 b) [37].

### Cytoskeletal elements

**Fig. 6:**
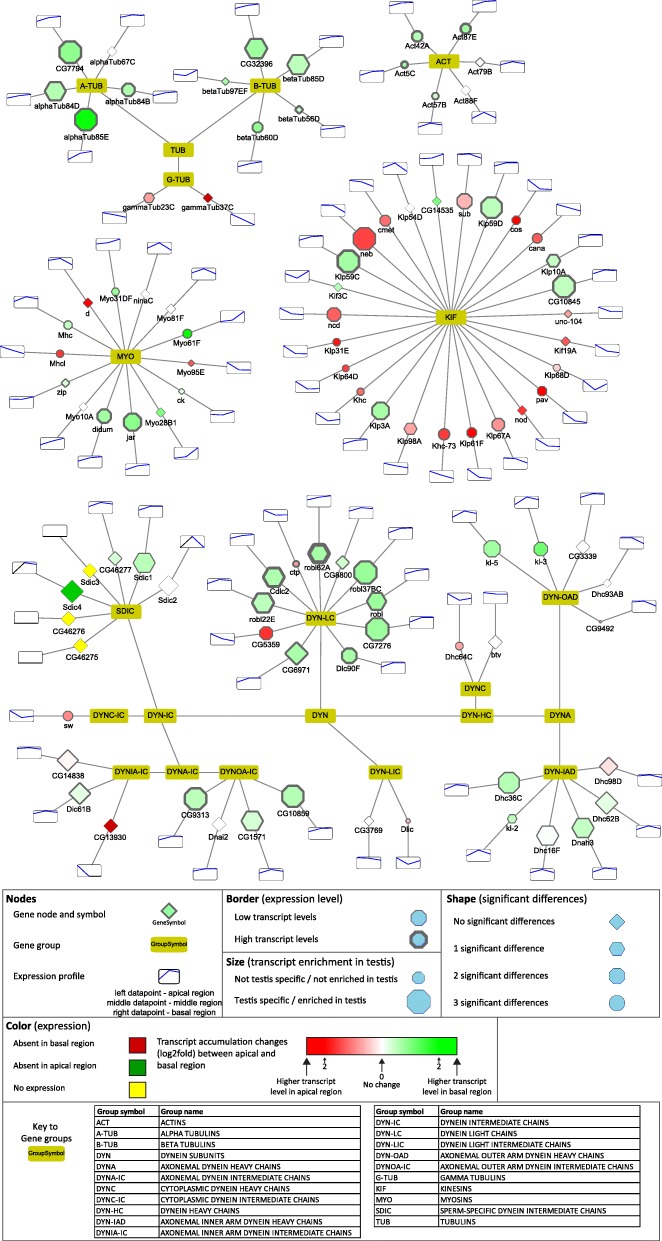
Distribution of transcripts of main cytoskeletal genes visualized by Cytoscape

Actin is the major component of the individualisation complex, forming the actin-based investment cones during the individualisation of the 64 cell cyst. While *Act42*, *Act87*, *Act57* and *Act5C* have moderately high transcript levels towards the basal region, *Act88F* and *Act79B* have very low transcript accumulation (Fig. 6).

Tubulins are the major components of mitotic spindles and the axoneme, so it is not surprising that their transcripts are present at all stages (Fig. 6). Gamma-tubulin transcripts accumulate in the apical region and their transcript level decreases towards the basal end of the testis (Fig. 6). All α- and β-tubulins show transcript accumulation toward the basal region, compared to the apical region, as expected from the major components of the axoneme (Fig. 6). Ovary-specific *αTub67C* has minimal transcript accumulation in the testis [38]. Different regions of the testis contain germ cells and also somatic cells, but our analysis is not able to distinguish between these cell types. *αTub85E* is known to localize specifically to the somatic cyst cells of the testis, which surround the developing spermatid cyst [39]. *αTub85E* and *βTub60D* are testis-specific tubulins with considerable transcript accumulation in the basal region, where they presumably contribute to the cytoskeletal composition of one of the largest somatic cell type of the fly. Interestingly, both alpha- and beta-tubulin families have further related members, *CG7794* and *CG32396* with transcript accumulation in the basal region of the testis, but the function of these genes must be clarified (Fig. 6).

Microtubule binding proteins, such as dyneins and kinesins, have essential functions in spermatogenesis. The SDIC family represents a sperm-specific dynein family, with four members. Depletion of SDIC genes resulted in normal fertility, but caused a reduction in sperm competition [40]. This result suggests that apart from the SDIC family, there are other dyneins involved in axonemal development. Our analysis shows that the transcripts of axonemal outer and inner arm dynein intermediate chains (*Dic61B*, *CG9313*, *CG10859*, *CG1571*) accumulate at the late stages, suggesting late function of these proteins (Fig. 6). The dynein light chain family is represented by numerous members with high transcript accumulation in the basal region, such as the Robl family, *Robl22E*, *Robl62A* and *Robl37BC* and several uncharacterized genes. We verified *CG5359* and *CG7276* by in situ hybridization and found *CG5359* RNA mainly at the apical and *CG7276* mainly towards the basal region of the testis (Fig. 2 C).The axonemal heavy chain family is also represented with apically- (*dhc98D*, *CG9492*) and basally- (*Dnah3*, *Dhc36C*, *kl-3*, *kl-5*) accumulating members (Fig. 6).

Five of the plus-end-directed kinesin motor proteins (*Klp59C*, *Klp59D*, *CG10845*, *Klp10A*, *Klp3A*) show accumulation in the basal region of the testis, while several of them are restricted to the spermatogonial stages of the apical region (Fig. 6). Functions of kinesin motor proteins are well-studied in male meiosis, influencing spindle assembly, chromosome segregation and centriole length, but the post-meiotic function of this protein family has yet to be elucidated [41, 42].

### Protein phosphorylation

An essential role of protein phosphorylation and dephosphorylation has been demonstrated during mitotic and meiotic divisions of spermatogenesis, however the function of these modifications is not well understood in the later post-meiotic stages or in the somatic cells of the testis. Our analysis identified several kinases with high testis-specificity and either apical or basal accumulation (Additional file 1: Figure S3), including the recently characterised Lkb1, which is important during individualisation [43]. Similarly, we identified several testis-enriched phosphatases with transcript accumulation in the basal region, suggesting intensive dephosphorylation activity after meiosis, either in somatic or germline cells of the testis (Additional file 1: Figure S4). It was shown that several members of the PPP family are strongly male-specific, however only PpY-55A is known to be localised to the nucleus of cyst cells, but the target molecules of these enzymes have yet to be revealed [44, 45]. Our analysis identified testis-specific members of almost all of the phosphatase families with transcript accumulation either in the apical or the basal region (Additional file 1: Figure S4). The precise biological role of these phosphatases during spermatogenesis, however, has to be defined.

### HSP-s

**Fig. 7:**
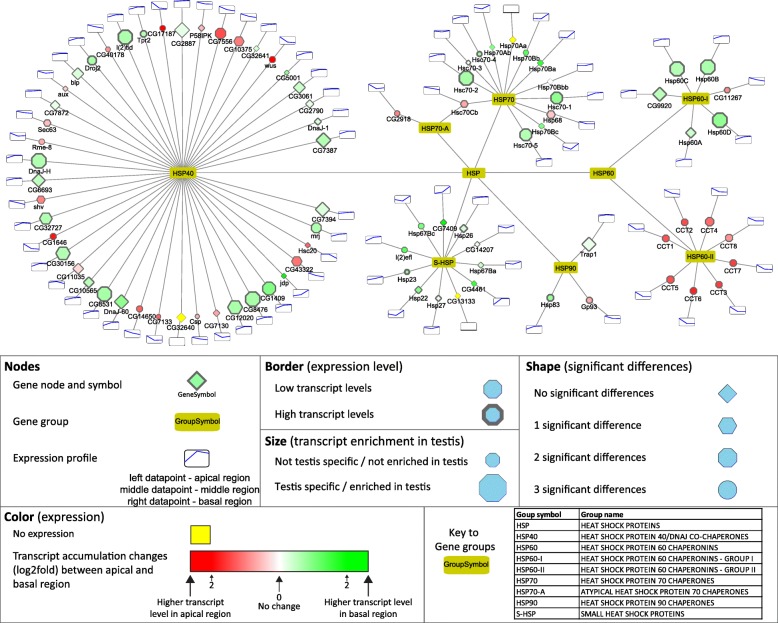
Distribution of transcripts of genes of the Hsp family visualized by Cytoscape

The main function of Hsp proteins is to prevent the accumulation of misfolded proteins. The Hsp gene group is represented by seven families, with numerous members in each family (Fig. 7). The Hsp60 family has two subfamilies, Hsp60-II is mainly present apically and Hsp60-I has mainly basally-enriched transcripts (Fig. 7). Hsp60B. from the Hsp60-I subfamily. is known to be required in the late stages of spermatogenesis in the individualisation of the 64 cells cyst [46]. Proteomic analysis identified twelve Hsp proteins in isolated sperm, suggesting important functions even in the mature sperm [47, 13]. The biggest member of the Hsp family is the Hsp40 group, with several exhibiting abundant apical and basal accumulation (Fig. 7). We tested the transcript distribution of two uncharacterized Hsp40 family genes, the apically enriched *CG7556* and the basally enriched *CG1409* by in situ hybridization and confirmed the RNA-Seq results (Fig. 2 E). Hsp40 family members play co-chaperone roles with several other Hsp proteins, such as Hsp70, which is involved in the development of mouse spermatogenic cells [48]. Hsp70–2 is known to be necessary for DNA packaging of spermatid nucleus in mice [49]. Small heat shock proteins are present in our analysis with examples of intermediate and high transcript accumulation patterns and three members have significant transcript differences toward the basal region. This is not surprising, because proteomic analysis of the sperm also revealed that the small heat shock protein Hsp67Bc is a member of the sperm proteome [13]. Despite the high number of different Hsp transcripts in the basal region, there is limited information about their biological roles.

### Metabolic pathways

**Fig. 8:**
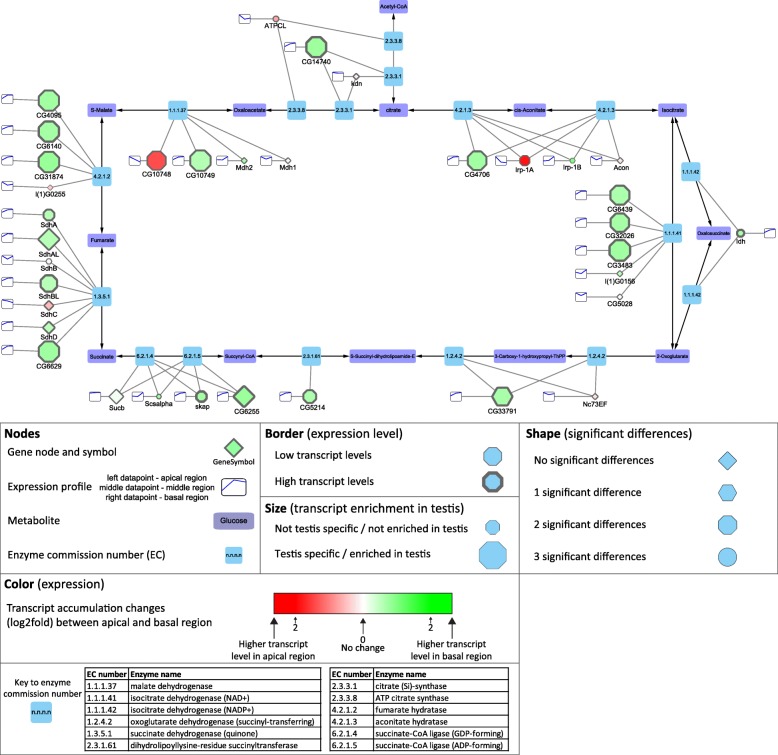
Distribution of transcripts of genes of the citrate cycle visualized by Cytoscape

Most of the genes encoding mitochondrial proteins are located in the nucleus in most contemporary eukaryotes, and many of them have duplicates with a testis-biased expression pattern for at least one paralogue [50, 51]. Based on the KEGG database, we reconstructed the central metabolism and the mitochondrial electron transport chain (oxidative phosphorylation) elements of *Drosophila melanogaster* using Cytoscape (Additional file 1: Figure S5, S6) [52]. We found that most of the metabolic enzymes of the citric acid cycle have one or more testis-specific counterparts with intensive accumulation in the basal part of the testis, where the elongated mitochondria are present (Fig. 8). We selected two succinate dehydrogenases from the citrate cycle and tested their transcript distribution in testis. *SdhC* is ubiquitously expressed gene, which shows apical enrichment in testis, while *CG6629* gene is testis-specific and its transcript accumulates mainly in the basal region, where post-meiotic cysts are present (Fig. 2 g, Fig. 8). This result suggests that late stages of spermatogenesis operate with a different set of mitochondrial enzymes than the cells in the apical region of the testis or other somatic cells of the body. Good examples for this are the glutamate dehydrogenases, where *gdh* is expressed in all somatic tissues of the fly, while *bb8,* a testis-specific paralogue, is expressed exclusively in testis [51]. A similar pattern was found for *Irp-1A* and *CG4706*, coding for aconitate hydratase. The *Irp-1A* transcript accumulates in the apical part, where mitotic spermatocytes are present, while transcripts of *CG4706* accumulate in the basal end, where the elongated spermatids are enriched. We also found that, besides the two ubiquitously expressed L-malate dehydrogenases, there are two testis-specific L-malate dehydrogenases, *CG10748* with apical and *CG10749* with basal enrichment (Fig. 2 G and Fig. 8). To elucidate the distribution of these enzymes during spermatogenesis, the tagged versions of the proteins were examined. CG10748-GFP localizes to the mitochondria during the early stages, accumulates in the nebenkern of the round spermatids and the protein is present until the elongation of spermatids. In contrast CG10749-mCherry localizes exclusively to the post-meiotic mitochondria, confirming the data of transcript distribution and also suggesting a spatially restricted function of the L-malate dehydrogenases during different stages of spermatogenesis (Fig. 2h). Similarly, to the citrate cycle, members of the electron transport chain of oxidative phosphorylation are represented by several testis-enriched genes with accumulation either in the apical or the basal regions (Additional file 1: Figure S6, Fig. 2 d). These results highlight that there are many testis-specific metabolic enzymes, which could serve the energy production of the specialized mitochondria in late stages of spermatogenesis and in the mature sperm.

### Long non-coding RNAs

Comprehensive analysis of long non-coding RNAs (lncRNA) revealed them as major players in a large number of pathways across species [53]. Out of the identified 2061 lncRNAs transcript, 203 lncRNAs show significant differences between apical and basal region of testis. We found 38 lncRNAs with significantly lower and 167 lncRNAs with significantly higher transcript level between the apical and the basal regions of the testis (Additional file 1: Figure S7). Moreover, our RNA-Seq results are also consistent with the recently published paper, where the authors suggest post-meiotic function for most of the tested lncRNAs [54]. We confirmed the region-specific distribution of four uncharacterized lncRNAs (*CR44076*, *CR44308*, *CR45622*, *CR46011*) by in situ hybridization (Fig. 2 f). These results suggest important functions of lncRNAs in post-meiotic spermatid development. It has to be elucidated, which of them are essential, what the degree of redundancy is between them and how they function at different developmental stages or in different tissues.

## Discussion

The field of transcriptomics using RNA-Seq has developed rapidly with the introduction of next-generation sequencing technologies and has replaced the microarray analysis method for gene expression profiling. RNA-Seq has enabled the detailed profiling of samples from very small amounts of starting material, such as single early embryonic stem cells [55]. The high sensitivity of RNA-Seq has enabled the identification of alternative splice variants and long non-coding RNAs in the transcriptome of the studied tissue. The modENCODE project highlighted that the majority of the *Drosophila melanogaster* genome is differentially expressed to generate a wide spectrum of protein-coding and non-coding RNAs at different developmental stages, which produces a transcriptome of unexpected complexity through the lifetime of the fly [56]. Importantly, expression of both protein-coding and non-coding regions are strongly male-biased, suggesting specific transcriptional regulation of sexual dimorphism and germ cell formation in males [56].

Microarray data and also whole testis RNA-Seq analysis are available to identify the testis-specific genes [7, 57]. Our work provides a comprehensive analysis of transcript distribution through the apical, middle and basal regions of the testis, which represent the main stages of spermatogenesis. The apical region is enriched with somatic and germ-line stem cells, cysts with primary and secondary spermatocytes. The middle region contains the 64 meiotic haploid round spermatids and the basal region has the elongated spermatids. Elongation, individualisation and the functionality of the sperm mainly rely on transcription and transcript accumulation before meiosis. Importantly, post-meiotic transcription is restricted to only a limited number of loci [58]. Regulation of protein synthesis from the synthetized RNA pool and degradation of proteins during individualisation is strictly controlled in space and time [59]. The central effectors of the ubiquitin proteasome system and the activation of the non-apoptotic function of caspases are responsible for protein degradation during individualisation [27, 36]. Both ubiquitination and deubiquitination are required for proper individualisation of the spermatids and several members of the process were recently identified [60, 61]. One example is caspase activation, which is regulated by the CRL3 ubiquitin ligase complex [62]. Active CRL3 associates with dBruce and the mitochondrial succinyl-CoA synthetase β subunit (A-Sβ) in the region of the individualisation complex and the inactive complex with Soti towards the tail. Soti inhibits caspase activity in spermatids from the proximal to distal region, restricting proteolytic degradation around the individualisation complex [22]. Our results highlight several members of the ubiquitination machinery with high transcript accumulation in the basal region, suggesting that there could be more players in the late degradation machinery. It was recently shown that metabolic and structural function of the citric acid cycle components A-Sβ are uncoupled. Metabolic enzymes on the surface of the mitochondria connect CRL3 containing E3 complexes for active degradation of cytoplasmic material during individualisation [62]. A deubiqutination enzyme, DUBA, is also necessary for individualisation and activation of caspase activity [35]. This suggests that there is an intimate connection and a fine regulation between mitochondria and the cytosol during the non-apoptotic degradation of cytoplasmic material during individualisation. In the mammalian system, Usp30 deubiquitinase was shown to localize to mitochondria and antagonize the ubiquitin ligase Parkin-mediated mitophagy [63]. Drosophila Parkin is essential in mitochondrial morphogenesis during spermatogenesis, so it will be interesting to test whether Usp30 has any connection in Parkin-mediated mitochondrial development [61].

Towards the basal end of the testis, we detected elevated transcript levels of the testis-specific subunits of almost every catalytic and regulatory particle of the proteasome, which provides candidates to identify as new members of the ubiquitin-proteasome system that play role in spermatogenesis. Based on the predominantly nuclear localisation of Prosα6T, a testis-specific subunit of the catalytic part of the proteasome, a major requirement of the proteasome for nuclear degradation, was suggested, which results in normal nuclear condensation and histone-to-protamine transition [37]. It was recently shown that the 19S subunit of the 26S proteasome also regulates the spreading of heterochromatin in yeast [64]. The majority of the testis-specific proteasome is composed of 20S proteasome, which binds to PA200, a protein essential for the acetylation-mediated degradation of core histones during mouse spermatogenesis [65]. Further experiments are necessary to clarify the precise composition and role of testis-specific proteasomal activity in nuclear compaction after meiotic stages in Drosophila spermatogenesis.

Cysts are covered by two somatic cyst cells, which grow simultaneously with the elongation of the spermatids. One of them covers the nuclear part of the cyst and the other one covers the 64 elongated 1,8 mm long tails, resulting an extremely large cell. Cyst cells and germ cells have an intimate connection, but there is relatively little information available about the transcript composition of the huge cyst cells. Our analysis cannot distinguish between the transcriptome of cyst cells and germ cells, therefore transcripts from cyst cells are represented in the dataset too. The results of a cyst cell-specific RNAi screen demonstrated that there is a cyst cell-specific gene network for each main stage of spermatogenesis. It was shown that a specific set of microtubules and the dynein-dynactin complex contribute to the morphological changes during elongation of the tail cyst cell [66]. We also identified both the known germ line-specific and cyst cell-specific cytoskeletal components as basally accumulating transcripts, which reflects the reliability of our approach.

The lifetime of RNAs and proteins influence their function, so the protein-coding genes with low transcript levels in the late stages could be important when the protein remains stable into the late stages of spermatogenesis. For example, *cut up*, a dynein light chain coding gene is transcribed in early stages, but the protein localises to the actin cones of the individualisation complex of spermatids [67]. However, both protein-coding genes and lncRNAs with high transcript accumulation in the basal region could represent the molecular components of spermatid development and the mature sperm. Genes with pleiotropic function during spermatogenesis could have transcripts in all examined regions, so we did not select them as significantly changing ones, but Additional file 2 contains the information about the expression of all examined genes.

Increasing evidence suggests that gene duplication can contribute to the specialized function of the post-meiotic mitochondrial proteins in Drosophila testis [68]. Our analysis identified transcript accumulation of the testis-specific counterpart of the citrate cycle and components of the oxidative phosphorylation in the basal region, where the specialized mitochondrial derivatives are present. Our results suggest that the specialisation of the mitochondria is completed by the expression of testis-specific paralogues of mitochondrial enzymes. Good examples for this are the testis-specific glutamate dehydrogenase (Bb8), the malate-dehydrogenase (CG10749), Cyt-C or a testis-specific ATP synthase gene with a specialized testis-specific mitochondrial function and expression pattern [51, 60]^,^ [69]. Further experiments are necessary to identify the molecular components of the elongated mitochondria, such as the paracrystalline material which fills the major mitochondrial derivative. In addition, the mechanism and place of energy production in the specialized mitochondria awaits further examination.

We found a surprisingly high number of lncRNAs with high testis specificity and basal enrichment. Recently, it was shown that the knock down of lncRNAs in the testis frequently resulted in late spermatogenesis defects, which may indicate their regulatory function at the final stages of spermatogenesis [54]. The fact that many of the newly evolved lncRNA genes expressed in the testis [70], and our analysis highlighted that most of them have maximal transcript levels in the basal region, further support the mainly unknown, but probably important function of lncRNA during the late stages of spermatogenesis or in the mature sperm.

## Conclusions

Our analysis provides a more detailed transcript composition of the coding and non-coding genes in the main stages of spermatogenesis and we were able to highlight the very special transcript composition of the basal region of testis, which contain the post-meiotic stages, where specialized protein degradation, cytoskeletal, nuclear or mitochondrial rearrangements and organelle specialization happen. Our searchable dataset and visualization method also offer a list of functionally related genes for further study, to gain a better understanding of different aspects of Drosophila spermatogenesis.

## Methods

### Isolation of tissue sample

Sample preparation followed Vibranovski et al. (Additional file 1: Figure S1) [7]. In brief, we used Oregon-R as a wild-type strain. Testes without the seminal vesicles were dissected in PBS on ice and we separated the apical (200), the middle (150) and the basal (150) region of testes with 0.25 mm diameter insect pins and transferred them into microcentrifuge tubes, which were frozen by liquid nitrogen after every 10 testes. Total RNA was prepared from the isolated and pooled testis region samples with ReliaPrep RNA Miniprep Sytem (Promega) following the recommendations of the manufacturer.

### RNA-Seq

RNA quality was verified by capillary gel electrophoresis in a Bioanalyzer 2100 instrument (Agilent) using the Agilent RNA 6000 Nano Kit, then RNA concentrations were determined by fluorometric measurement with a Qubit 2.0 fluorometer (Thermo Fisher Scientific) using the Qubit RNA HS Assay kit. Indexed RNA-Seq libraries were prepared from 800 ng total RNA using the TruSeq RNA Library Prep Kit v2 (Illumina) following the TruSeq RNA Sample Prep v2 LS protocol provided by the manufacturer. This, in short, includes purification of poly(A) mRNA with oligo-dT magnetic beads, RNA fragmentation, synthesis of double-stranded cDNA using SuperScript II reverse transcriptase (Invitrogen), ligation of indexed Illumina adapters and amplification with limited-cycle PCR. Sequencing libraries were validated by capillary electrophoresis using the Agilent DNA 1000 kit in a Bioanalyzer 2100 instrument, then quantitated with the Qubit dsDNA HS Assay Kit in a Qubit 2.0 instrument and with the KAPA Library Quantification Kit (KAPA Biosystems) in a Piko-Real Real-Time PCR System (Thermo Fisher Scientific). 4 nM sequencing libraries were pooled, denatured with 0.1 M NaOH and, after dilution, paired-end sequencing was done with an Illumina MiSeq sequencer using the MiSeq Reagent Kit V3–150. Primary sequence analysis was done by BaseSpace cloud computing environment. FastQ files were aligned to the Dmr6.05 *Drosophila melanogaster* reference genome using TopHat v2.0.9., then differential gene expression analysis was done using Cuffdiff v2.1.1. The selection of differentially accumulated transcripts was based on the pairwise comparison of the three tested regions of testis. Genes with significant differences (*p*-values are less than 0.05, after correction for multiple comparisons with Benjamini-Hochberg method) were regarded as differentially expressed. The false-discovery rate corrected p-values and the log_2_ fold changes of the expression data are provided in Additional file 2.

### Bioinformatical analysis

Tissue enrichment was calculated based on the work of Li et al. [11]. We calculated the tissue specificity of gene expression in testes using the modENCODE database. To utilize log2 fold changes for the visualization, we used the apical region and basal region datasets. To eradicate the extreme values caused by values between 0 and 1, we rounded up the whole dataset, and re-calculated the log2-fold changes. Gene groups data were acquired from FlyBase (FB2017_05 release), metabolic pathways were reconstructed based on the KEGG database. Male-biased genes (3720) were selected based on the SeBiDA database [10] to test the correlation of 3015 genes from our normalised sequencing data and the normalised microarray data from the work of Vibranovski et al. [7]. We normalised the data as follows: x_n_ = (x_i_-x̄)/σ where x_n_ is the normalised value, x_i_ the original value, x̄ is the average of the dataset and σ is the standard deviation of the dataset. For each comparative analysis, we updated the related gene IDs from FlyBase. GO enrichment analysis was conducted on GOrilla [14, 71]. *P* value threshold was 10^^− 3^. Ranked lists were created based on testis specificity index, FPKM in apical, middle, and basal regions with higher values at the top. We created target lists based on the resolved testis specificity, differences in transcript levels between the apical and the basal regions. Target lists of genes were searched for GO enrichment on the background of the whole dataset. Graphs were created in Microsoft Excel. Networks were built in Cytoscape 3.5.1 [15]. Line charts were created with the Enhanced Graphics plugin of Cytoscape [72].

### RNA in situ hybridization

cDNA was produced with RevertAid First Strand cDNA Synthesis Kit (Thermo Fisher Scientific) based on the manufacturer’s instructions and used to amplify ~ 500 bp long templates. Probes were synthesized with DIG RNA Labelling Kit (SP6/T7) (Roche) according to the manufacturer’s instructions. In situ hybridization was performed as described by White-Cooper with the following differences: hybridization buffer contains the additional 100 μg ml^− 1^ tRNA (Sigma) [73]. Images were taken by using Olympus BX51 microscope.

### Molecular cloning and microscopy of CG10748-GFP and CG10749-mCherry constructs

Genomic DNA was purified from 30 flies and was used for PCR reaction with Phusion high fidelity DNA polymerase (New England Biolabs). CG10748-GFP and CG10749-mCherry constructs were designed and constructed by using NEBuilder Hifi DNA assembly master mix (New England Biolabs) based on the manufacturer’s protocol. The constructs contain the 5′ regulatory element, the 5’ UTR region, the coding sequence of each gene and C-terminal GFP or mCherry sequence in a pUASTattB vector. Transgenic constructs were injected into flies containing a source of φ-31 integrase and an attP landing site (Bloomington Stock Center No: 25709). Testis preparation and staining were performed as earlier described by White-Cooper 2004 [73]. 4′,6-diamidino-2-phenylindole (DAPI) was used at 1 μg ml^− 1^ final concentration. Samples were mounted in Slowfade diamond antifade mountant (Thermo Fisher Scientific). Fluorescent images were taken by using Olympus Fluoview Fv10i Confocal microscope.

## Additional files

Additional file 1: **Figure S1.** Testis sample preparation and analysis of RNA-Seq results. **Figure S2.** Transcript distribution of the member of the ubiquitin system E3, and Cullin, SKP1 and F-Box genes visualized by Cytoscape. **Figure S3.** Distribution of transcripts of kinases visualized by Cytoscape. **Figure S4.** Distribution of transcripts of phosphatases visualized by Cytoscape. **Figure S5.** Distribution of transcripts of genes involved in citrate cycle and the sugar metabolism visualized by Cytoscape. **Figure S6.** Distribution of transcripts of genes of OXPHOS visualized by Cytoscape. **Figure S7.** Distribution of transcripts of long non-coding RNA genes visualized by Cytoscape. (PDF 12098 kb)

Additional file 2: Differential gene expression analysis, and testis specificity. Results of the differential gene expression analysis of different regions and the testis specificity index of Drosophila genes. (XLSX 2979 kb)

Additional file 3: GO analysis. Summary of different GO term analyses. (XLSX 257 kb)

Additional file 4: Full network of the analysed FlyBase Gene Group data visualised by Cytoscape. Cytoscape session file contains the whole gene group network of FB2017_05 FlyBase release, and the numeric values of each node related data. Cytoscape 3 session file (.cys). Appropriate viewer: Cytoscape 3 (http://www.cytoscape.org/). (CYS 1139 kb)

## Abbreviations

DmSP: *Drosophila melanogaster* sperm proteome
DUB: Deubiquitinases
FPKM: Fragments Per Kilobase per Million mapped reads
GO: Gene Ontology
lncRNA: Long non-coding RNA
UEV: Ubiquitin E2 enzyme variants
UPS: Ubiquitin Proteasome System
