# Supplementary material for: Analysis of Drosophila melanogaster testis transcriptome
Source: BMC Genomics. 2018 Sep 24;19:697. doi: 10.1186/s12864-018-5085-z (PMC6154878; doi:10.1186/s12864-018-5085-z)
Supplement: Supplementary file 1 — Figure S1. Testis sample preparation and analysis of RNA-Seq results. Figure S2. Transcript distribution of the member of the ubiquitin system E3, and Cullin, SKP1 and F-Box genes visualized by Cytoscape. Figure S3. Distribution of transcripts of kinases visualized by Cytoscape. Figure S4. Distribution of transcripts of phosphatases visualized by Cytoscape. Figure S5. Distribution of transcripts of genes involved in citrate cycle and the sugar metabolism visualized by Cytoscape. Figure S6. Distribution of transcripts of genes of OXPHOS visualized by Cytoscape. Figure S7. Distribution of transcripts of long non-coding RNA genes visualized by Cytoscape. (PDF 12098 kb) [file 12864_2018_5085_MOESM1_ESM.pdf]

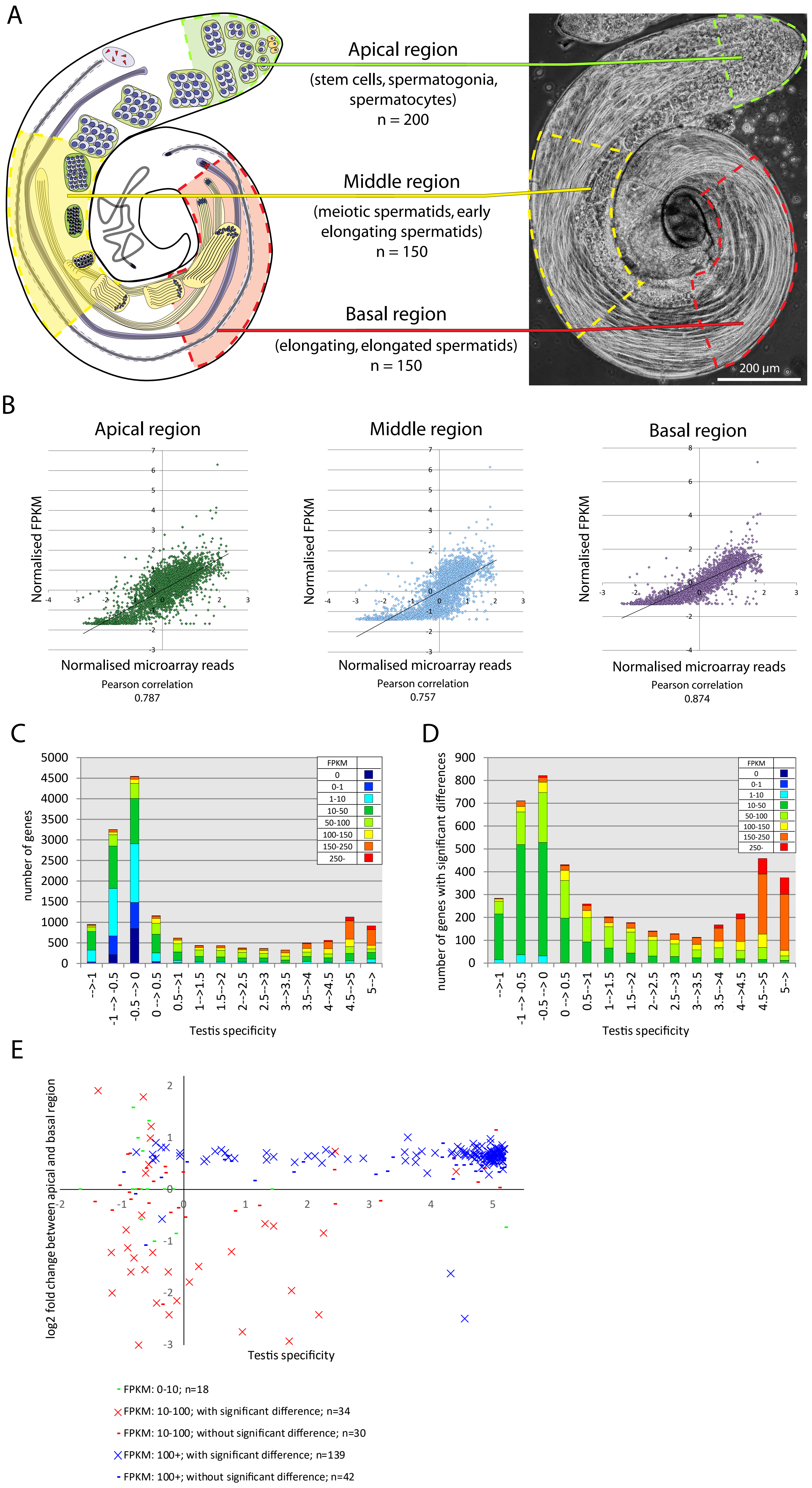

FigS1. **Testis sample preparation and analysis of RNA-Seq results**

(A) Schematic representation of the isolated apical, middle and basal testis regions with the indicated stages of spermatogenesis. (B) Charts represent the correlation of gene expression-based on normalised microarray data[6] and normalised RNA-Seq data of male-biased genes (Sebida database) in different regions of the testis (C) Transcript levels of genes are clustered based on the testis specificity index. (D) Transcript levels of genes showing significant expression changes are clustered based on the testis specificity index. (E) Scatter plot represents the log2 fold changes of transcript levels of genes of sperm proteome[11, 12] as a function of testis specificity index.

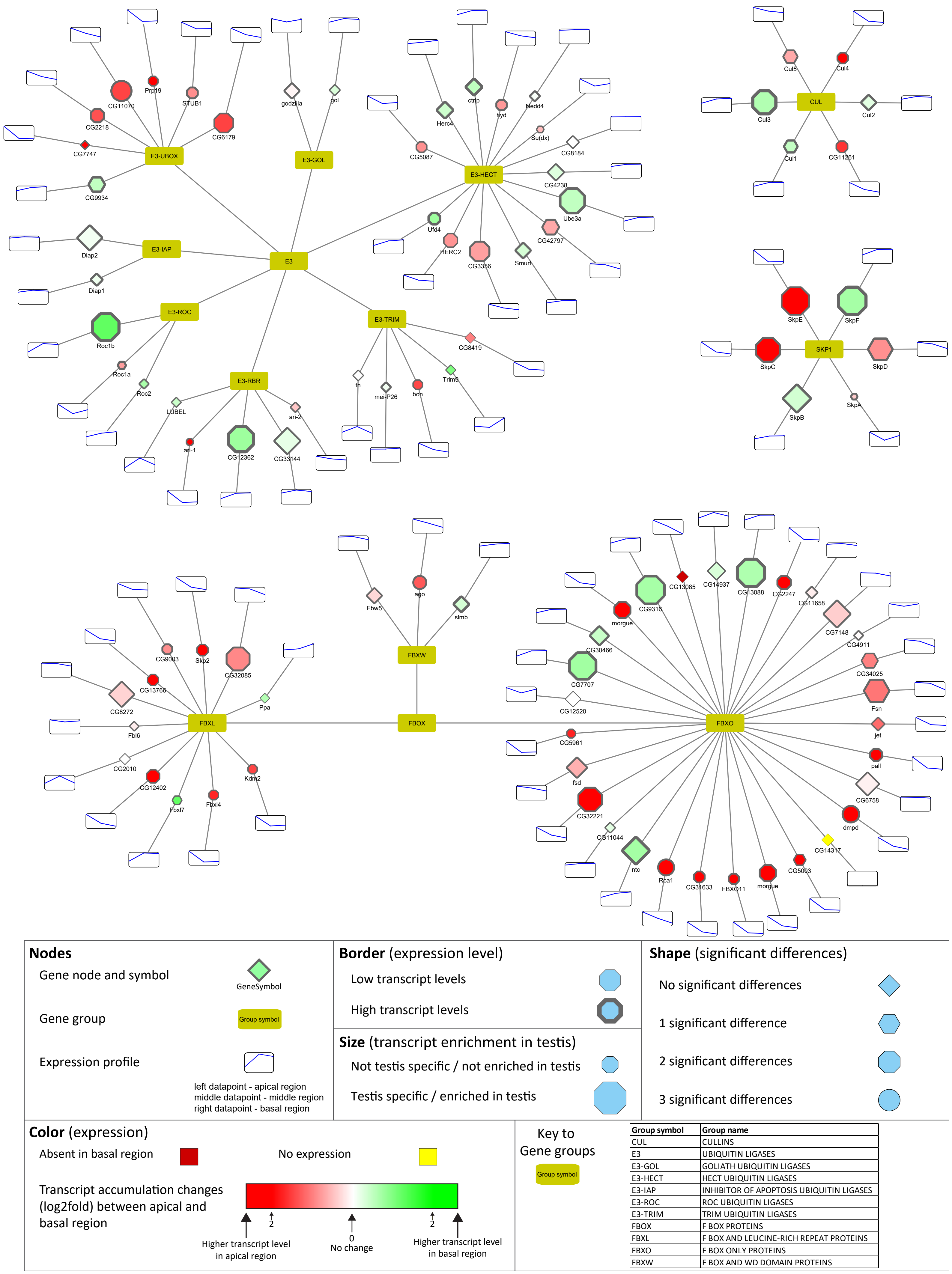

FigS.2. **Transcript distribution of the member of the ubiquitin system E3, and Cullin, SKP1 and F-Box genes visualized by Cytoscape.**

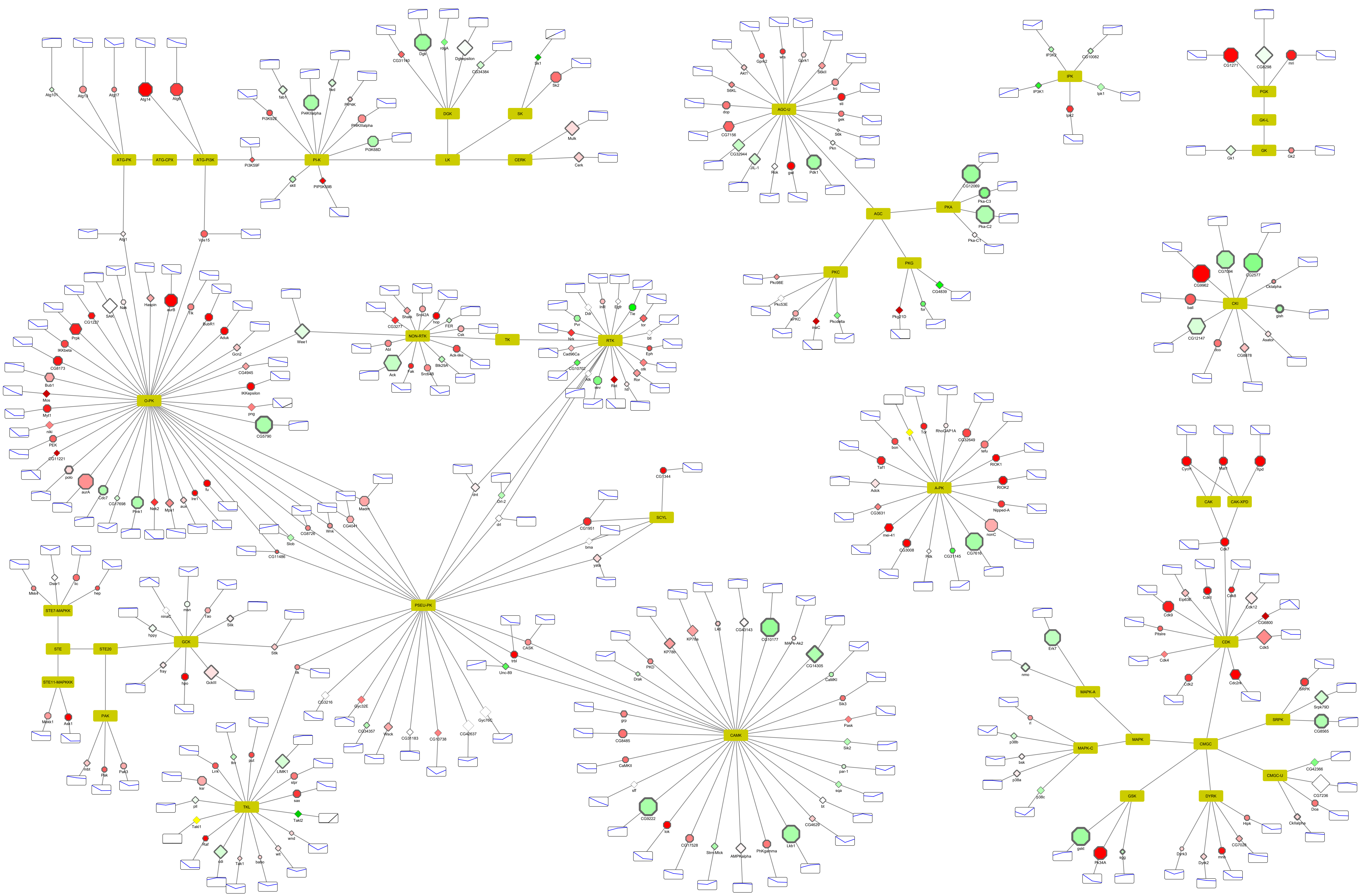

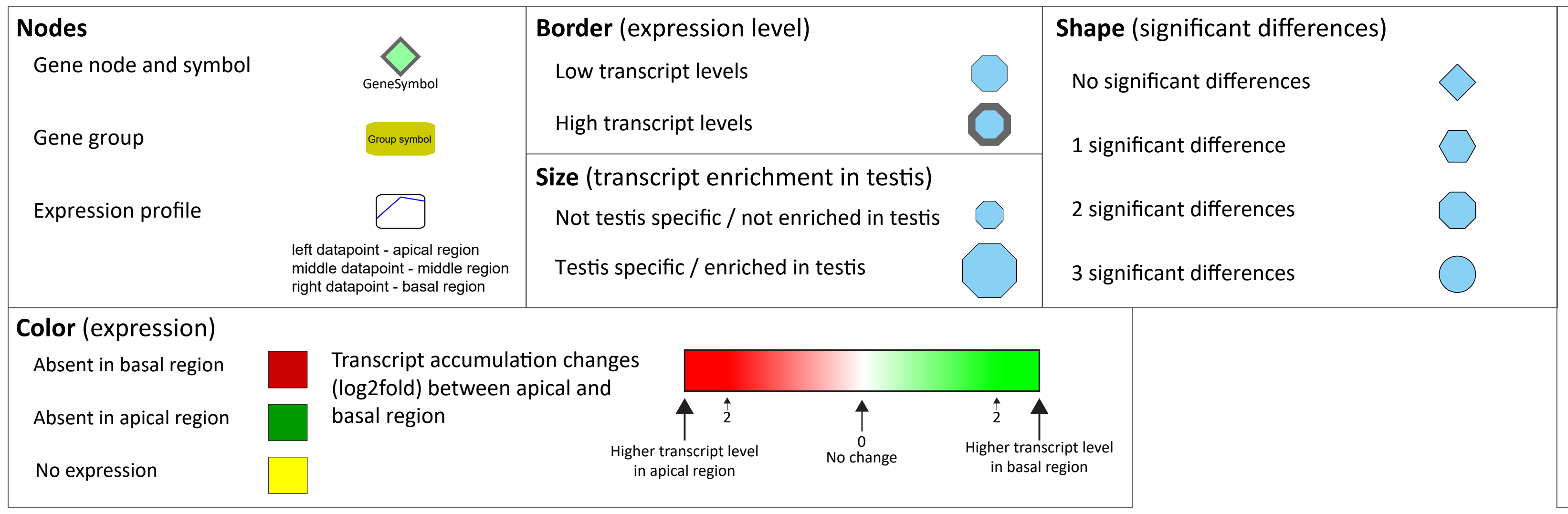

Key to Gene groups

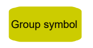

Group symbol	Group name
AGC	AGC-KINASES
AGC-U	AGC KINASES (UNCLASSIFIED)
A-PK	ATYPICAL PROTEIN KINASES
ATG-CPX	AUTOPHAGY-RELATED COMPLEXES
ATG-PI3K	AUTOPHAGY-SPECIFIC PHOSPHATIDYLINOSITOL 3-KINASE COMPLEX
ATG-PK	ATG1 PROTEIN KINASE COMPLEX
CAK	CDK-ACTIVATING KINASE COMPLEX
CAK-XPD	CDK-ACTIVATING KINASE-XERODERMA PIGMENTOSUM D COMPLEX
CAMK	CALCIUM/CALMODULIN-DEPENDENT PROTEIN KINASES
CDK	CYCLIN DEPENDENT KINASES
CERK	CERAMIDE KINASES
CKI	CK1 KINASES
CMGC	CMGC KINASES
CMGC-U	CMGC KINASES (UNCLASSIFIED)
DGK	DIACYLGLYCEROL KINASES
DYRK	DUAL SPECIFICITY TYROSINE-PHOSPHORYLATION REGULATED KINASES
GCK	GERMINAL CENTER KINASES
GK	GLYCEROL KINASE
GK-L	GLYCEROL KINASE-LIKE
GSK	GLYCOGEN SYNTHASE 3 KINASES
IPK	INOSITOL PHOSPHATE KINASES
LK	LIPID KINASES

Group symbol	Group name
MAPK	MITOGEN ACTIVATED PROTEIN KINASES
MAPK-A	ATYPICAL MITOGEN ACTIVATED PROTEIN KINASES
MAPK-C	CONVENTIONAL MITOGEN ACTIVATED PROTEIN KINASES
NON-RTK	NON-RECEPTOR TYROSINE KINASES
O-PK	OTHER CONVENTIONAL PROTEIN KINASE DOMAIN KINASES
PAK	P21-ACTIVATED KINASES
PGK	PSUEDO-GLYCEROL KINASE
PI-K	PHOSPHATIDYLINOSITOL KINASES
PKA	PROTEIN KINASE A FAMILY
PKC	PROTEIN KINASE C FAMILY
PKG	PROTEIN KINASE G FAMILY
PSEU-PK	PROTEIN PSEUDOKINASES (KNOWN AND PUTATIVE PSEUDOKINASES)
RTK	RECEPTOR TYROSINE KINASES
SCYL	SCYL PSEUDOKINASES
SK	SPHINGOSINE KINASES
SRPK	SERINE-ARGININE PROTEIN KINASES
STE	STE KINASES
STE11-MAPKKK	STE11 KINASES (MAPK KINASE KINASES)
STE20	STE20 KINASES
STE7-MAPKK	STE7 KINASES (MAPK KINASES)
TK	TYROSINE KINASES
TKL	TYROSINE KINASE-LIKE KINASES

FigS.3. **Distribution of transcripts of kinases visualized by Cytoscape.**

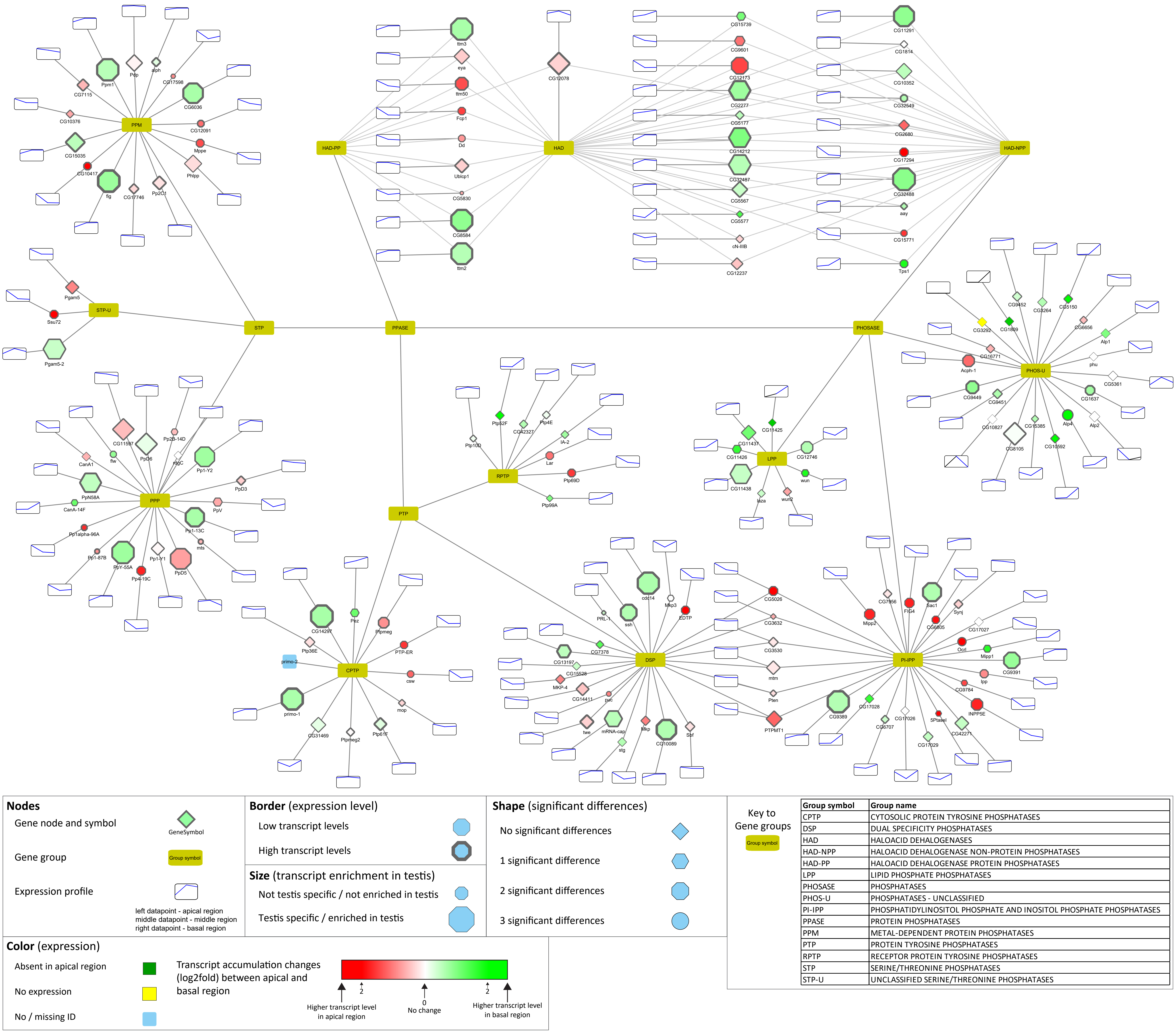

Group symbol	Group name
CPTP	CYTOSOLIC PROTEIN TYROSINE PHOSPHATASES
DSP	DUAL SPECIFICITY PHOSPHATASES
HAD	HALOACID DEHALOGENASES
HAD-NPP	HALOACID DEHALOGENASE NON-PROTEIN PHOSPHATASES
HAD-PP	HALOACID DEHALOGENASE PROTEIN PHOSPHATASES
LPP	LIPID PHOSPHATE PHOSPHATASES
PHOSASE	PHOSPHATASES
PHOS-U	PHOSPHATASES - UNCLASSIFIED
PI-IPP	PHOSPHATIDYLINOSITOL PHOSPHATE AND INOSITOL PHOSPHATE PHOSPHATASES
PPASE	PROTEIN PHOSPHATASES
PPM	METAL-DEPENDENT PROTEIN PHOSPHATASES
PTP	PROTEIN TYROSINE PHOSPHATASES
RPTP	RECEPTOR PROTEIN TYROSINE PHOSPHATASES
STP	SERINE/THREONINE PHOSPHATASES
STP-U	UNCLASSIFIED SERINE/THREONINE PHOSPHATASES

FigS.4. **Distribution of transcripts of phosphatases visualized by Cytoscape.**

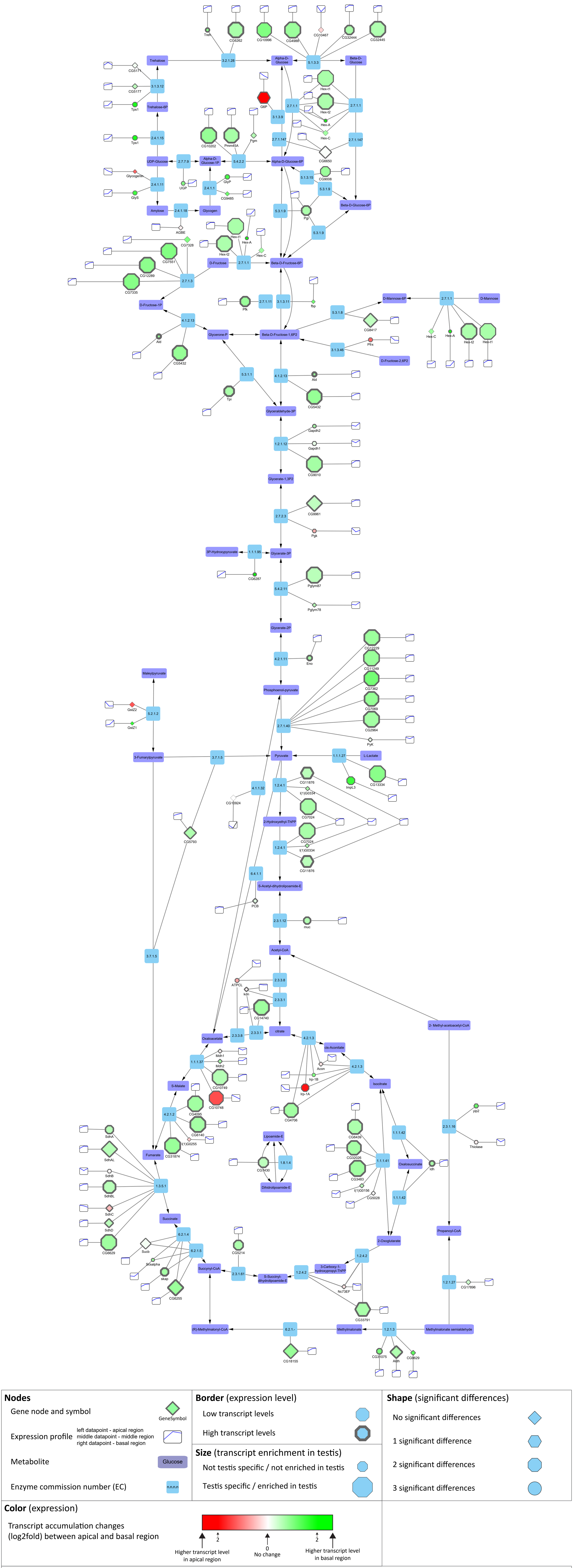

Key to enzyme commission number

n.n.n.n

EC number	Enzyme name
1.1.1.27	L-lactate dehydrogenase
1.1.1.37	malate dehydrogenase
1.1.1.41	isocitrate dehydrogenase (NAD+)
1.1.1.42	isocitrate dehydrogenase (NADP+)
1.1.1.95	phosphoglycerate dehydrogenase
1.2.1.12	glyceraldehyde-3-phosphate dehydrogenase (phosphorylating)
1.2.1.27	methylmalonate-semialdehyde dehydrogenase (acylating)
1.2.1.3	aldehyde dehydrogenase (NAD+)
1.2.4.1	pyruvate dehydrogenase (acetyl-transferring)
1.2.4.2	oxoglutarate dehydrogenase (succinyl-transferring)
1.3.5.1	succinate dehydrogenase (quinone)
1.8.1.4	dihydrolipoyl dehydrogenase
2.3.1.12	dihydrolipoyllysine-residue acetyltransferase
2.3.1.16	acetyl-CoA C-acyltransferase
2.3.1.61	dihydrolipoyllysine-residue succinyltransferase
2.3.3.1	citrate (Si)-synthase
2.3.3.8	ATP citrate synthase

EC number	Enzyme name
2.4.1.1	phosphorylase
2.4.1.11	glycogen(starch) synthase
2.4.1.15	a,a-trehalose-phosphate synthase (UDP-forming)
2.4.1.18	1,4-a-glucan branching enzyme
2.7.1.1	hexokinase
2.7.1.11	6-phosphofructokinase
2.7.1.147	ADP-dependent glucokinase
2.7.1.3	ketohexokinase
2.7.1.40	pyruvate kinase
2.7.2.3	phosphoglycerate kinase
2.7.7.9	glucose-1-phosphate uridylyltransferase
3.1.3.11	fructose-bisphosphatase
3.1.3.12	trehalose-phosphatase
3.1.3.46	fructose-2,6-bisphosphate 2-phosphatase
3.1.3.9	glucose-6-phosphatase
3.2.1.28	a,a-trehalase
3.7.1.5	acylpyruvate hydrolase

EC number	Enzyme name
4.1.1.32	phosphoenolpyruvate carboxykinase (GTP)
4.1.2.13	fructose-bisphosphate aldolase
4.2.1.11	phosphopyruvate hydratase
4.2.1.2	fumarate hydratase
4.2.1.3	aconitate hydratase
5.1.3.15	glucose-6-phosphate 1-epimerase
5.1.3.3	aldose 1-epimerase
5.2.1.2	maleylacetoacetate isomerase
5.3.1.1	triose-phosphate isomerase
5.3.1.8	mannose-6-phosphate isomerase
5.3.1.9	glucose-6-phosphate isomerase
5.4.2.11	phosphoglycerate mutase
5.4.2.2	phosphoglucomutase
6.2.1.-	Acid--thiol ligases
6.2.1.4	succinate-CoA ligase (GDP-forming)
6.2.1.5	succinate-CoA ligase (ADP-forming)
6.4.1.1	pyruvate carboxylase

FigS.5. **Distribution of transcripts of genes involved in citrate cycle and the sugar metabolism visualized by Cytoscape.**

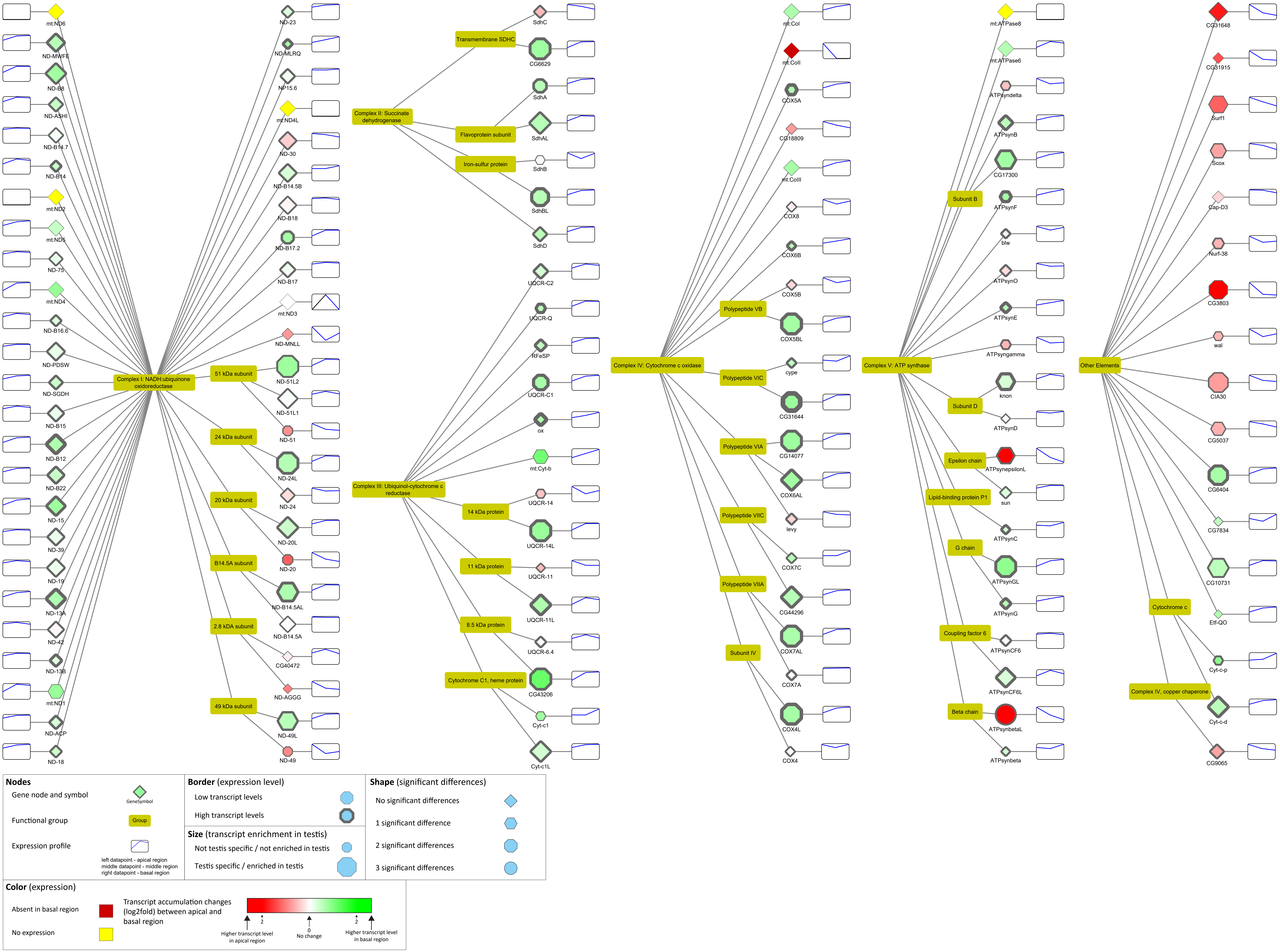

FigS.6. **Distribution of transcripts of genes of OXPHOS visualized by Cytoscape.**

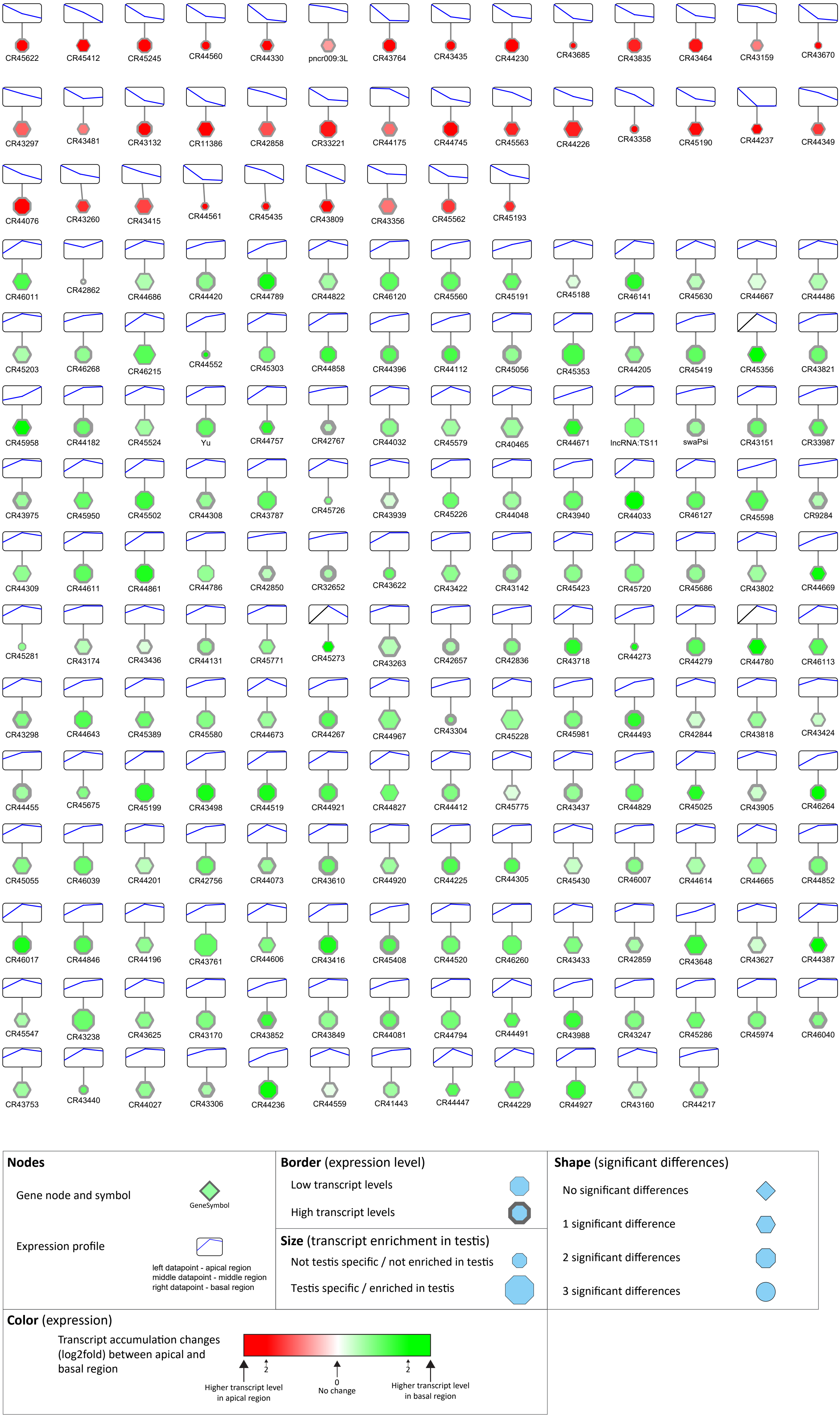

FigS.7. **Distribution of transcripts of long non-coding RNA genes with significant differences visualized by Cytoscape.**
